# COVID-19 Infection vs Vaccination and the Risk of New-onset Psoriasis

**DOI:** 10.2340/actadv.v106.44217

**Published:** 2026-01-08

**Authors:** Sophie L. PREUß, Henning OLBRICH, Katja BIEBER, Artem VOROBYEV, Eva Lotta MODEREGGER, Khalaf KRIDIN, Henner ZIRPEL, Diamant THAÇI, Evelyn GAFFAL, Ralf J. LUDWIG, Philip CURMAN

**Affiliations:** 1 Department of Dermatology, University Medical Center of the State of Schleswig-Holstein (UKSH), Campus Lübeck, Lübeck; 2 Lübeck Institute of Experimental Dermatology, University of Lübeck, Lübeck, Germany; 3 Azrieli Faculty of Medicine, Bar-Ilan University, Safed, Israel; 4 Unit of Dermatology and Skin Research Laboratory, Barch Padeh Medical Center, Poriya, Israel; 5 Institute and Comprehensive Centre for Inflammation Medicine, University of Lübeck, Germany; 6 Dermato-Venereology Clinic, Karolinska University Hospital, Stockholm; 7 Department of Medical Epidemiology and Biostatistics, Karolinska Institutet, Stockholm; 8 Dermatology and Venereology Division, Department of Medicine (Solna), Karolinska Institutet, Stockholm, Sweden

**Keywords:** psoriasis, COVID-19, vaccination

## Abstract

Emerging evidence has suggested a link between new-onset psoriasis and both COVID-19 infection and vaccination, though findings have been limited by small case numbers and lack of adequate control groups. This retrospective cohort study used electronic health records from the US Collaborative Network of TriNetX from January 2020 to January 2025 to compare the risk of developing new-onset psoriasis in individuals with confirmed COVID-19 infection and no vaccination history vs those vaccinated without prior infection. Propensity score matching was applied to balance demographics, comorbidities, and psoriasis risk factors. The primary outcome was a new diagnosis of psoriasis (ICD10-CM: L40.0–5) within 3 months following infection or vaccination. Subgroup analyses assessed the specific codes L40.0-5 separately. Kaplan–Meier survival analysis and Cox proportional hazards models were used to compare outcomes. Patients with COVID-19 infection had a significantly higher risk of developing psoriasis compared with vaccinated individuals (HR 1.30; 95% CI, 1.14–1.49; *p* < 0.001). Increased risks were also observed for psoriatic arthritis and pustulosis palmaris et plantaris. These findings suggest a potential triggering role of infection in psoriasis pathogenesis and support the safety profile of vaccination. Further studies are needed to confirm causality and guide clinical decision-making.

## SIGNIFICANCE

In this large, propensity score-matched cohort study, individuals with COVID-19 infection had a significantly higher risk of developing new-onset psoriasis within 3 months compared with those who received COVID-19 vaccination. These findings provide new, population-level evidence for a differential risk of psoriasis onset depending on the type of immune exposure. This study offers a novel perspective on the relationship between immune triggers and psoriasis onset. By directly comparing infection and vaccination, it provides an estimate of the relative frequency of psoriasis development following these exposures. These findings may support clinical understanding of psoriasis risk in the context of infection and vaccination.

The global COVID-19 pandemic has had a profound impact on healthcare systems worldwide, presenting significant challenges for both patients and healthcare providers (1). The rapid development and widespread distribution of COVID-19 vaccines have played a crucial role in mitigating the spread of the virus and preventing severe outcomes. Both COVID-19 infection and vaccination can act as immune system triggers, potentially influencing the course of autoimmune and immune-mediated diseases.

Psoriasis is a chronic inflammatory skin disorder with a well-established association with immune system triggers. The onset of psoriasis has been strongly linked to various infections, including the most commonly established, streptococcal infections (2), as well as other bacterial and viral infections (3). Simultaneously, associations with different vaccinations, such as influenza, BCG, and tetanus-diphtheria vaccines, have also been reported (4–7).

There are several types of COVID-19 vaccines, including mRNA vaccines (such as Pfizer-BioNTech and Moderna), viral vector vaccines (like AstraZeneca and Johnson & Johnson), and protein subunit vaccines (8). Numerous studies, case series, and reports have documented new-onset psoriasis and flare-ups following COVID-19 vaccination. A systematic review by Wu et al. summarized 11 of these studies on new-onset psoriasis (35 patients) and 36 studies on psoriasis flare-ups (279 patients) (9). Another systematic review by Potestio et al. included 49 studies with a total of 134 patients, of whom 27 developed new-onset psoriasis and 107 experienced psoriasis exacerbation (10). A recent systematic review by Karampinis et al. identified 71 patients with plaque psoriasis flares, 12 patients with new-onset psoriasis, and 17 patients with a change in plaque psoriasis subtype (11). However, the absence of comparison groups in these individual studies limits the ability to draw robust conclusions from the observed associations.

Regarding COVID-19 infection, several reports indicate an elevated risk of developing autoimmune and chronic inflammatory skin diseases, including psoriasis (12–15). However, the comparative risk of developing psoriasis after COVID-19 infection vs COVID-19 vaccination remains insufficiently explored.

This study aims to address this critical knowledge gap by evaluating the risk of psoriasis following COVID-19 infection compared with COVID-19 vaccination. Utilizing real-world data from the TriNetX Analytics Network, it seeks to provide valuable insights into the potential associations between these exposures and the development of psoriasis.

## MATERIAL AND METHODS

### Study design and data source

**Fig. 1:**
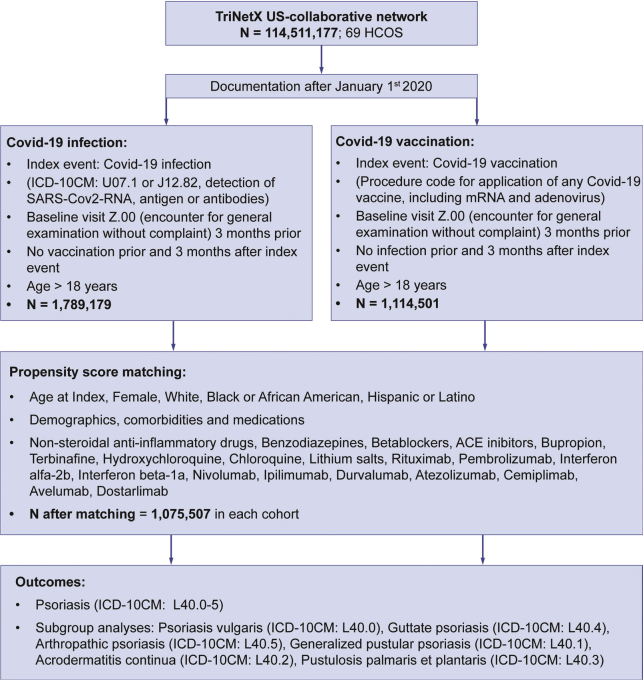
**Flowchart depicting the study outline.** Electronic health records (EHR) were accessed from the United States Collaborative Network database of TriNetX. EHRs with documentation of COVID-19 infection or vaccination after 1 January 2020 and aged 18 or older were included. Extensive 1:1 propensity-score matching was performed. Groups were compared with each other by risk and survival analyses.

A large-scale propensity-score matched retrospective cohort study using electronic health record (EHR) data from TriNetX (https://trinetx.com/), a global federated healthcare database and platform, was performed (16, 17). Similar previously published protocols were regarded (15). The TriNetX platform provides secure, de-anonymized, real-time access to EHRs from several healthcare organizations (HCOs) on the global level. In this study, data from the US Collaborative Network, totalling over 113 million EHRs, was accessed, retrieved, and analysed in January 2025. Two cohorts were defined (detailed below): (1) COVID-19 infection and (2) COVID-19 vaccination. The index event, determining the start of the study period and entry of each participant in the study, was defined as an instance of COVID-19 infection or COVID-19 vaccination, respectively **(****Fig. 1****)**. To capture the period of the COVID-19 pandemic, only EHRs documented since 1 January 2020 were included.

### Study population definitions

Two cohorts were defined: (1) 1,790,539 individuals with COVID-19 infection and (2) 1,088,207 individuals with COVID-19 vaccination. Both cohorts were defined using a combination of ICD-10CM, LOINC, SNOMED, CPT, and TNX curated codes indicating either COVID-19 infection (ICD-10CM: U07.1 or J12.82) or detection of SARS-CoV-2 RNA, antigen, or antibodies, or documentation of any form of COVID-19 vaccine or immunization event **(**Table SI). In the infection group, all forms of vaccination were excluded from any time before index until the end of the study period. Similarly, in the vaccination group, all forms of COVID-19 infection were excluded similarly. To further balance the 2 cohorts and provide a fair comparison, all participants were drawn from the same pool of eligible individuals by forcing an occurrence of ICD-10CM:Z00 (“Encounter for general examination without complaint, suspected or reported diagnosis”) at any time at least 3 months before index. Only individuals 18 years and older at index were considered.

### Covariates

Extensive propensity-score matching (PSM) was performed to reduce confounding by balancing both cohorts for known and potential confounding factors. A total of 42 covariates, including demographics (e.g., age at index, sex, race), somatic diseases, psychiatric disorders, and risk factors for psoriasis such as nicotine dependence, obesity, streptococcal infections, and medications were included in the PSM (Table SII**)**. In sensitivity analysis S4 with altered cohort definitions (see below), a limited number of covariates (*n* = 12) was used due to computational limitations as a result of the larger total cohort size.

### Outcomes and analyses

ICD-10CM codes were used to define the study outcomes. As primary outcome a composite outcome “any psoriasis” (L40.0-5) was defined, excluding unspecified/other psoriasis codes (e.g., L40.8–9). For subgroup analysis the outcomes were: psoriasis vulgaris (L40.0), guttate psoriasis (L40.4), arthropathic psoriasis (L40.5), generalized pustular psoriasis (L40.1), and acrodermatitis continua (L40.2). The data were coded and collected in the United States. In the ICD-10CM system, code L40.3 is a subcategory of L40 (Psoriasis) and is specifically defined as pustulosis palmaris et plantaris (PPP). The term “PPP“ is used inconsistently across authors and regions and remains a subject of ongoing controversy, particularly regarding its distinction from palmoplantar pustular psoriasis and whether it represents 1 entity as psoriasis-related disease or a localized form of pustular psoriasis. As the ICD-10CM system does not further differentiate, code L40.3 reflects a broader, inclusive approach to classification within ICD-10CM (18–24).

The primary analysis investigated outcomes between 7 and 90 days after index. The short time window was chosen to precisely capture acute immune reactions and reduce the influence of confounders. It is also in line with previously published literature reporting time until onset after vaccination (25). To evaluate the robustness of findings across varying follow-up lengths, we performed additional sensitivity analyses with shorter windows: S1 (7–30 days), S2 (7–45 days), and S3 (7–60 days), progressively extending the follow-up time. S4 removed the forced Z00 requirement to increase cohort size, and S5 ensured follow-up by requiring a post-index healthcare visit after 1 week to 6 months, with the exclusion of any deceased patient due to the increased mortality rates in COVID-19. Note that any outcomes during the first week weres removed in all analyses to mitigate potential immediate detection bias.

### Statistical considerations

Logistic regression analysis was used to generate a propensity score for each patient (with exposure as the dependent variable) using Python (https://www.python.org/) and the scikit-learn package (https://scikit-learn.org/). PSM was performed 1:1 using the greedy nearest neighbour approach with a cut-off distance of 0.1 pooled standard deviations of the logit of the propensity score. The matrix row order was randomized after data retrieval. Differences in baseline characteristics for continuous variables were compared by *t*-test and for binary or categorical variables by z-test. Survival analyses were performed using Kaplan–Meier (KM) analysis. The proportional hazards assumption was tested by the coxph function in R’s Survival package (R Foundation for Statistical Computing, Vienna, Austria) using Schoenfeld residuals and χ^2^ tests. KM curves were compared using the log-rank test; *p*-values were adjusted for multiple testing by the Holm-Šidák correction. A univariate Cox proportional hazards regression was used to express hazard ratios (HR)s with 95% confidence intervals (CIs). Any individuals with outcomes prior to the index event were excluded in all analyses, with exclusion taking place after PSM. Analyses were performed on the TriNetX platform.

### Ethics statement

TriNetX contains solely anonymized data that is presented only in aggregated form, complying with the de-identification standard as defined by the US Health Insurance Portability and Accountability Act (HIPAA) in section §164,514(a). All data access and analysis took place behind firewalls on the secure servers of the participating HCOs. TriNetX is certified to the ISO 27001:2013 standard and maintains an Information Security Management System to ensure rigorous protection of all data. The use of the TriNetX platform for research was approved by the Swedish Ethical Review Authority (diary number 2025-03805-02).

## RESULTS

### Study population characteristics

Data from a total of 113,055,731 patients from 67 US HCOs were screened. The number of study participants after successful PSM were 1,075,507 each for the infection and vaccination cohorts (see Fig. 1). Baseline characteristics and all matching variables, derived from the primary analysis, are listed in **Table I**. Differences in matching covariates were considerably reduced after PSM. The mean follow-up times between compared cohorts were similar for all comparisons (data not shown).

**Table I:** Baseline characteristics for the primary analysis (1 week–3 months)

Characteristic	Before matching			After matching		
	COVID-19 infection	COVID-19 vaccination	SD	COVID-19 infection	COVID-19 vaccination	SD
Number of participants	1,789,179	1,114,501	–	1,075,507	1,075,507	–
Demographics						
Age at Index (years, SD)	46.5±22.5	54±18.8	0.364	54.2±19.2	53.5±18.8	0.041
Female (*n*)	1,029,642	588,628	0.095	585,637	575,865	0.018
White (*n*)	1,209,556	718,455	0.066	720,685	706,050	0.029
Black or African American (*n*)	268,627	125,520	0.111	122,209	124,871	0.008
Hispanic or Latino (*n*)	158,074	158,074	0.041	98,868	104,771	0.019
Diagnoses						
Overweight and obesity (*n*)	504,225	313,976	0.001	314,301	305,722	0.018
Nicotine dependence (*n*)	189,700	131,670	0.038	126,651	126,276	0.001
Streptococcus group A infections (*n*)	2,682	1,329	0.008	1,256	1,303	0.001
Family history of skin diseases (*n*)	3,007	1,350	0.012	1,197	1,338	0.004
Endocrine, nutritional and metabolic diseases (*n*)	1,156,928	786,751	0.127	769,549	755,165	0.029
Cardiovascular diseases (*n*)	886,579	625,790	0.132	607,894	599,652	0.015
Respiratory system diseases (*n*)	1,190,534	638,215	0.192	648,796	630,026	0.036
Genitourinary system diseases (*n*)	1,009,629	644,589	0.028	642,230	622,458	0.037
Digestive system diseases (*n*)	1,042,954	652,778	0.006	636,395	630,530	0.011
Blood-related diseases (*n*)	497,034	307,125	0.005	306,719	299,325	0.015
Infections (*n*)	804,976	430,845	0.129	430,779	423,097	0.015
Neoplasms (*n*)	573,383	444,064	0.163	425,411	420,562	0.009
Musculoskeletal system and connective tissue diseases (*n*)	1,209,927	789,406	0.069	780,359	760,231	0.042
Nervous system diseases (*n*)	932,531	611,609	0.055	606,455	589,854	0.031
Injuries and poisoning (*n*)	947,068	550,139	0.071	571,351	533,553	0.070
Mental, behavioural, and neurodevelopmental disorders (*n*)	871,597	549,359	0.012	546,000	530,696	0.028
External causes of morbidity (*n*)	517,519	296,974	0.051	310,815	287,932	0.047
Medications						
Non-steroidal anti-inflammatory drugs (*n*)	911,575	541,348	0.048	535,542	526,179	0.017
Benzodiazepines (*n*)	651,849	466,078	0.111	450,624	445,021	0.011
Betablockers (*n*)	441,681	304,543	0.060	298,918	294,161	0.010
ACE inhibitors (*n*)	292,777	227,913	0.106	217,199	216,389	0.002
Bupropion (*n*)	122,511	79,133	0.010	78,514	76,934	0.006
Terbinafine (*n*)	36,993	27,391	0.026	25,743	26,078	0.002
Hydroxychloroquine (*n*)	24,489	14,258	0.008	14,128	14,011	0.001
Lithium salts (*n*)	6,936	5,133	0.011	4,831	4,873	0.001
Rituximab (*n*)	6,857	4,031	0.004	4,079	3,972	0.002
Pembrolizumab (*n*)	1,509	1,193	0.007	1,126	1,155	0.001
Interferon beta-1a (*n*)	1,179	806	0.002	758	789	0.001
Chloroquine (*n*)	1,013	789	0.006	714	749	0.001
Nivolumab (*n*)	792	666	0.007	622	632	0.000
Ipilimumab (*n*)	347	307	0.005	264	295	0.002
Durvalumab (*n*)	292	225	0.003	229	213	0.001
Atezolizumab (*n*)	258	214	0.004	206	204	0.000
Interferon alpha-2b (*n*)	197	168	0.004	145	157	0.001
Cemiplimab (*n*)	40	30	0.001	32	26	0.001
Avelumab (*n*)	33	21	0.000	25	21	0.001
Dostarlimab (*n*)	10	10	0.001	10	10	0

SD: standard differentiation.

### Increased risk of psoriasis following COVID-19 infection compared with vaccination

The incidence of the primary outcome “any psoriasis” was higher after COVID-19 infection (0.046%) than after vaccination (0.035%). The hazard ratio was 1.30 (95% CI: 1.14–1.49; adjusted *p* < 0.001), indicating a significantly increased risk following infection.

When examining specific psoriasis subtypes, arthropathic psoriasis (L40.5) showed the highest risk difference in patients with infection compared with vaccination, with an HR of 1.44 (1.18–1.77) and an adjusted *p*-value of < 0.001. Additionally, PPP (L40.3) demonstrated a statistically significant increased risk in patients with infection compared with vaccination (HR 1.34, 1.05–1.71).

**Fig. 2:**
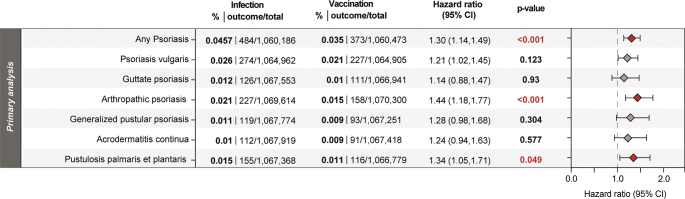
**Association of COVID-19 infection with increased risk of psoriasis, arthropathic psoriasis,, and pustulosis palmaris et plantaris compared with COVID-19 vaccination.** Patients with documented COVID-19 infection or vaccination were compared with each other by risk and survival analyses after propensity-score matching. The total numbers of patients identified, as well as the observed risk (in %), are shown. Bars represent hazard ratios (HR) with confidence intervals, adjusted *p*-values of log-rank tests are shown.

Trends for increased risks of other psoriasis subtypes were observed, albeit not statistically significant: psoriasis vulgaris (L40.0) (HR 1.21, 1.02–1.45), generalized pustular psoriasis (L40.1) (HR 1.28, 0.98–1.68), guttate psoriasis (L40.4) (HR 1.14, 0.88–1.47), and acrodermatitis continua (L40.2) (HR 1.24, 0.94–1.63) **(Fig. 2)**.

### Consistently elevated risk of psoriasis after COVID-19 infection compared with vaccination across different follow-up windows

In sensitivity analysis 1 (follow-up 1 week–1 month), patients with COVID-19 infection had a significantly higher risk of any psoriasis (HR 1.56, 1.22–2.00), arthropathic psoriasis (HR 1.73, 1.19–2.52), and PPP (HR 1.85, 1.19–2.86). No significant differences were found for other subtypes.

Also in sensitivity analysis 2 (follow-up 1 week–1.5 months), the risk of any psoriasis remained higher in patients with infection (HR 1.40, 1.15–1.70), with significant increases for arthropathic psoriasis (HR 1.48, 1.10–1.99) and PPP (HR 1.86, 1.31–2.65) compared with patients with vaccination. Also the risk for generalized pustular psoriasis (HR 1.67, 1.13–2.46) was higher in patients with infection compared with vaccination in this follow-up period.

In sensitivity analysis 3 (follow-up 1 week–2 months), infection was associated with a significantly higher risk of any psoriasis (HR 1.40, 1.19–1.65), arthropathic psoriasis (HR 1.46, 1.14–1.88), and PPP (HR 1.80, 1.32–2.45) but also generalized pustular psoriasis (HR 1.71, 1.21–2.42) and acrodermatitis continua (HR 1.65, 1.16–2.33).

In sensitivity analysis 4 (without baseline visit), the risk of any psoriasis was higher in infection (HR 1.45, 1.32–1.61), with significant increases for psoriasis vulgaris (HR 1.37, 1.20–1.57), arthropathic psoriasis (HR 1.60, 1.39–1.84), generalized pustular psoriasis (HR 1.32, 1.07–1.62), and PPP (HR 1.331.11–1.59).

**Fig. 3:**
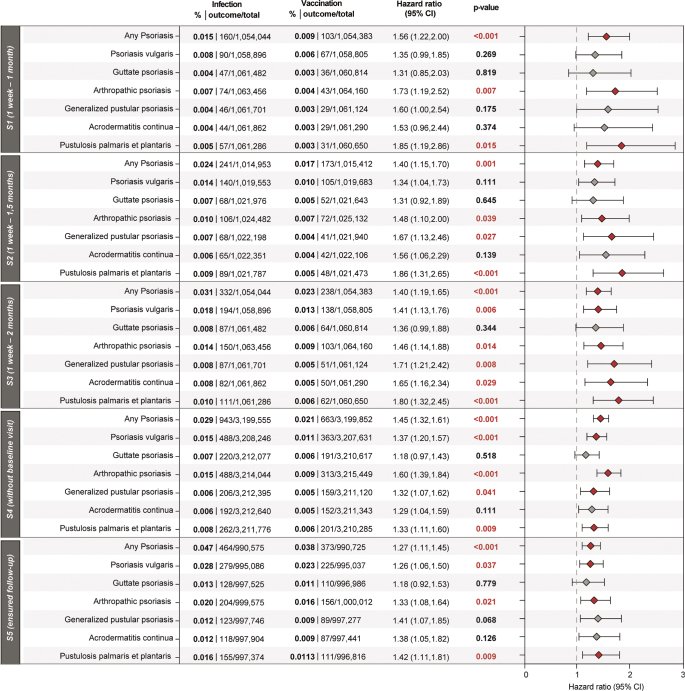
**Higher risks of psoriasis following COVID-19 infection compared with COVID-19 vaccination confirmed by five sensitivity analyses.** To evaluate the study’s robustness, 5 different sensitivity analyses were incorporated (S1: outcomes 7–30 days, S2: outcomes 7–45 days, S3: outcomes 7–60 days, S4: outcomes 7–30 days with removal of forced ICD-10CM:Z00 prior to index, increasing cohort sizes more than threefold, and S5: outcomes 7–30 days with ensured follow-up, defined by the documentation of at least 1 healthcare visit 1 week to 6 months after index, with the exclusion of any deceased patient due to the increased mortality rates in COVID-19). Significant risk differences were observed for “any psoriasis”, arthropathic psoriasis, and pustulosis palmaris et plantaris. Total numbers of patients at risk identified, as well as observed risks in percentages are shown. Bars represent hazard ratios (HR) with confidence intervals; adjusted *p*-values of log-rank tests are shown.

In S5 (ensured follow-up), infection was associated with a significantly higher risk of any psoriasis (HR 1.27, 1.11–1.45), psoriasis vulgaris (HR 1.26, 1.06–1.51), arthropathic psoriasis (HR 1.33, 1.08–1.64), and PPP (HR 1.42, 1.11–1.81) **(Fig. 3)**.

## DISCUSSION

This is the first large-scale study comparing the risk of new onset of psoriasis following COVID-19 infection compared with COVID-19 vaccination. A significantly elevated risk to develop the composite endpoint psoriasis, as well as the subgroups PPP and arthropathic psoriasis, was observed after infection compared with vaccination. These risks persisted in all sensitivity analyses.

The incidence of psoriasis in the general population of the United States was 0.064% per year, which corresponds to approximately 0.016% over 3 months (26). In our study, the overall onset rate within 3 months was 0.046% in the infected cohort and 0.035% in the vaccinated cohort.

The higher risk of developing psoriasis after a COVID-19 infection compared with vaccination could be attributed to the stronger and often dysregulated immune response triggered by the infection. COVID-19 infection can lead to a hyperinflammatory state and cytokine storm, which are characterized by the excessive release of pro-inflammatory cytokines such as IL-1ß, IL-6, TNF-α, and IL-17 (27–30). These cytokines are known to play a central role in the pathogenesis of psoriasis (31).

In contrast, COVID-19 vaccines induce a more targeted immune response that, while capable of causing inflammatory reactions, is generally less intense and more controlled than the immune response elicited by natural infection (32, 33). Serum levels of IL-6, TNF-α, and IL-8 were significantly lower in vaccinated individuals compared with those who had recovered from severe COVID-19 infection (34).

We found a significantly increased risk of PPP after COVID-19 infection compared with vaccination in all sensitivity analyses. Previously, there have been reports concerning the new onset of pustular dermatoses after COVID-19 infection (14, 35). However, there was no significantly increased risk of generalized pustular psoriasis in the infection compared with the vaccination group.

COVID-19 infection has previously been reported to be associated with psoriatic arthritis flares (36–38). Similarly, the more robust immune activation induced by infection as opposed to vaccination may contribute to the reactivation of inflammatory pathways in predisposed individuals.

In this study, we chose to compare individuals with documented COVID-19 infection but no vaccination with those who received a COVID-19 vaccination and had no documented infection. A third group without either exposure was deliberately not included as we considered it unlikely that a substantial portion of the population remained entirely unexposed to SARS-CoV-2 during the pandemic. Asymptomatic or mild infections frequently went undetected and unrecorded, making it difficult to reliably define a truly unexposed group.

It is also important to acknowledge that individuals in the vaccination group may have experienced unrecorded COVID-19 infections that were not captured in the data. However, any such infections were likely mild or asymptomatic, as more severe cases would typically be coded. Thus, the vaccination group may be more accurately characterized as representing individuals who were vaccinated, with or without concurrent mild infection. This limitation should be considered when interpreting the findings, particularly regarding the attribution of risk specifically to vaccination. If anything, such misclassification would likely result in the true risk differences being even greater.

In general, it is important to recognize that incomplete recording of both infections and vaccinations, particularly early in the pandemic, is an inherent limitation of real-world data. However, this effect is likely to be at least partially compensated by the large sample size.

Importantly, our findings do not establish causality. It is possible that individuals who chose to be vaccinated differed systematically from those who became infected. We sought to minimize this risk of confounding through extensive propensity-score matching, which included not only known risk factors for psoriasis but also a broad range of comorbidities, medication, and external factors of morbidity.

It must also be accounted for that individuals with acute infections, which are potential triggers of psoriasis,, might have had COVID-19 vaccination deferred, which could introduce selection bias towards healthier individuals in the vaccinated cohort. Moreover, the classification of psoriasis subtypes relies on diagnostic coding, which may be subject to inaccuracies or inconsistencies in clinical practice. The severity of psoriasis was not possible to assess as the TriNetX database includes diagnostic codes and visit-related data only, without validated severity measures. Further limitations of the study include potential residual confounding and the observational nature of the data, which inherently carries biases.

Together, limitations of the TriNetX database include variability in diagnostic coding across participating healthcare organizations, the inability to verify the accuracy or completeness of recorded diagnoses, and the lack of information on care received outside contributing institutions, as well as differences in healthcare-seeking behaviour. Additionally, clinical details such as laboratory parameters and disease activity measures are not systematically captured, limiting the granularity of clinical characterization.

A major strength of our study is the use of a large, real-world cohort. Extensive propensity-score matching and multiple sensitivity analyses were employed to minimize confounding and ensure the stability of the results. Additionally, a stringent definition of psoriasis was used, excluding codes for other and unspecified types. Importantly, this study addresses a highly relevant clinical question: as COVID-19 infection and vaccination continue to impact global populations, understanding their differential effects on chronic inflammatory diseases such as psoriasis is critical for patient counselling, risk assessment, and public health strategies.

In summary, this is the first large-scale study directly comparing the risk of new-onset psoriasis following COVID-19 infection vs vaccination. Our results suggest that vaccination may not only protect against COVID-19 infection itself but could also potentially reduce the risk of chronic inflammatory diseases such as psoriasis that may be triggered by infection.

Future observational studies and meta-analyses are warranted to confirm these observations and to further investigate the immunopathological mechanisms underlying the differential risks associated with infection and vaccination.
